# *SCN5A* Mutations in Brugada Syndrome Are Associated with Increased Cardiac Dimensions and Reduced Contractility

**DOI:** 10.1371/journal.pone.0042037

**Published:** 2012-08-02

**Authors:** Frans van Hoorn, Maria E. Campian, Anje Spijkerboer, Marieke T. Blom, R. Nils Planken, Albert C. van Rossum, Jacques M T. de Bakker, Arthur A M. Wilde, Maarten Groenink, Hanno L. Tan

**Affiliations:** 1 Department of Radiology, Academic Medical Center, University of Amsterdam, Amsterdam, The Netherlands; 2 Department of Heart Failure Research Center, Academic Medical Center, University of Amsterdam, Amsterdam, The Netherlands; 3 Department of Cardiology, VU University Medical Center, Amsterdam, The Netherlands; 4 Department of Cardiology, Academic Medical Center, University of Amsterdam, Amsterdam, The Netherlands; Goethe University, Germany

## Abstract

**Background:**

The cardiac sodium channel (Na_v_1.5) controls cardiac excitability. Accordingly, *SCN5A* mutations that result in loss-of-function of Na_v_1.5 are associated with various inherited arrhythmia syndromes that revolve around reduced cardiac excitability, most notably Brugada syndrome (BrS). Experimental studies have indicated that Na_v_1.5 interacts with the cytoskeleton and may also be involved in maintaining structural integrity of the heart. We aimed to determine whether clinical evidence may be obtained that Na_v_1.5 is involved in maintaining cardiac structural integrity.

**Methods:**

Using cardiac magnetic resonance (CMR) imaging, we compared right ventricular (RV) and left ventricular (LV) dimensions and ejection fractions between 40 BrS patients with *SCN5A* mutations (*SCN5a-*mut-positive) and 98 BrS patients without *SCN5A* mutations (*SCN5a-*mut-negative). We also studied 18 age/sex-matched healthy volunteers.

**Results:**

*SCN5a-*mut-positive patients had significantly larger end-diastolic and end-systolic RV and LV volumes, and lower LV ejection fractions, than *SCN5a-*mut-negative patients or volunteers.

**Conclusions:**

Loss-of-function *SCN5A* mutations are associated with dilatation and impairment in contractile function of both ventricles that can be detected by CMR analysis.

## Introduction

The cardiac sodium channel, Na_v_1.5, controls cardiac excitability by triggering the action potential of working cardiac myocytes and driving electric impulse transmission. Its pore-forming α-subunit is encoded by *SCN5A*. Accordingly, *SCN5A* mutations that result in loss-of-function of Na_v_1.5 are associated with various inherited arrhythmia syndromes that revolve around reduced cardiac excitability (“loss-of-function *SCN5A* channelopathy”) [1]. The most prevalent syndrome is Brugada syndrome (BrS) [2]. More rarely, loss-of-function *SCN5A* mutations cause progressive cardiac conduction disease [3], atrial standstill [4], atrioventricular (AV) block [5], or sinus node disease [6]. It has long been assumed that cardiac structural abnormalities are undetectable by clinical imaging methods in individuals with loss-of-function *SCN5A* channelopathies. This would be consistent with the conventional concept that Na_v_1.5 is only involved in maintaining electrical integrity of the heart. However, this paradigm has been challenged by the recent discovery that Na_v_1.5 may also be involved in maintaining structural integrity of the heart. Although unexpected, such an involvement was supported by experimental studies which have indicated that Na_v_1.5 is part of a macromolecular complex that contains cystoskeleton/cytoskeleton-associated proteins (reviewed in [7]). Moreover, loss-of-function *SCN5A* mutations were found in rare patients with dilated cardiomyopathy [8]. Still, clinical evidence to link *SCN5A* mutations to structural derangements is anecdotal [9]–[11], equivocal [12]–[13], or indirect [14]–[16]. For instance, while one study showed histopathological derangements in myocardial biopsies of BrS patients [12], the pathophysiologic role of these derangements was questioned in another study [13]. Studies of BrS patients that used cardiac magnetic resonance (CMR) imaging [14]–[17] showed subtle abnormalities, including reduced contractile function of both right ventricle (RV) and left ventricle (LV), and dilatation of the RV outflow tract (RVOT). Yet, these studies were not designed to analyze whether such changes are related to the presence of *SCN5A* mutations, as they did not specifically compare patients with *SCN5A* mutations to patients without *SCN5A* mutations (BrS is also linked to other genes, and *SCN5A* mutations, while playing an important role in BrS, are only found in up to 25% of BrS patients [18]). Such an analysis would address the question whether *SCN5A* variants *per se* cause structural cardiac derangements.

With the aim of obtaining clinical evidence whether *SCN5A* is involved in maintaining cardiac structural integrity, we systematically compared cardiac dimensions and contractility between 40 BrS patients with *SCN5A* mutations and 98 BrS patients without *SCN5A* mutations using cardiac magnetic resonance imaging (CMR).

## Methods

This single-center study was conducted according to the principles expressed in the Declaration of Helsinki. The Ethics Committee of the Academic Medical Center Amsterdam approved this study. Written informed consent was obtained from all patients and controls.

### CMR Analysis

We studied 138 consecutive *SCN5A* genotyped BrS patients (57 probands) who had undergone CMR. We studied 3 groups: (1) patients with *SCN5A* mutations (*SCN5A*-mut-positive, n = 40), (2) patients without *SCN5A* mutations (*SCN5A*-mut-negative, n = 98), (3) age/sex-matched healthy volunteers (n = 18). We used a 1.5 Tesla whole-body imaging system (Avanto, Siemens, Germany) with a dedicated phased-array cardiac coil. The heart was visualized in the standard long axis and short axis views, the latter encompassing the total heart, using standard available steady state free precession sequences. Scan parameters were: TR 1-2ms, TE 2-4ms, Flip Angle 60–80°, slice thickness 6 mm, spatial resolution in the x-y direction: 1–2 mm/pixel, temporal resolution 20–30 ms. To evaluate the presence of myocardial fatty infiltration and edema, we acquired short axis double inversion T1 weighed and T2 weighed, fat saturated black blood images, encompassing the total heart (TR = RR interval, TE 40 ms, slice thickness 6 mm, interslice gap 2 mm, spatial resolution in the X-Y plane ≥1 mm/pixel). After administration of intravenous Gadolinium contrast, additional double inversion T1 weighed black blood images with fat-suppression to assess late enhancement (as an indicator of myocardial fibrosis) was acquired in the axial direction. Images were acquired during repeated end-expiratory breath-holds. Quantitative analysis was performed off-line using dedicated commercially available software (MASS, Leiden, The Netherlands). LV and RV end-systolic and end-diastolic images were isolated in the stack of short axis CINE images and the endocardial borders were delineated manually. From end-systolic and end-diastolic LV and RV volumes, calculated by the modified Simpson’s rule, stroke volumes and ejection fractions were derived. Thickness of the LV posterior wall and anterior interventricular septum (IVS) were measured on an end-diastolic short-axis slice immediately basal to the tips of the papillary muscles. RVOT area was measured at the level of the aortic valve on the axial black blood images. Dimensions were corrected for body surface area (BSA). All image analyses were performed by two experienced observers who were blinded to the clinical history and results of genetic screening.

### Molecular Genetic Analysis

The entire coding region of *SCN5A* was analyzed as described previously [19]. Truncating mutations were defined as those in which a premature stop codon was present or caused by a frameshift. In all *SCN5A*-mut-negative patients, we also screened *SCN1B* and *GPD1-L*, but found no mutations.

### Statistical Analysis

Data are mean±SD. Two-tailed t-test was performed to compare group means, Chi-square test to compare proportions. Linear regression analysis was performed to study the relation between ECG parameters (heart rate, PR duration, QRS duration) and cardiac dimensions. Linear regression analysis was also performed to compare CMR data between patient groups, thereby correcting for sex and presence of coronary artery disease. p<0.05 was considered statistically significant.

## Results

### Demographic and ECG Data

Demographic data were not different between groups (Table 1). Consistent with our previous study [20], *SCN5A*-mut-positive patients had evidence of generally slower conduction than *SCN5A*-mut-negative patients, i.e., longer electrocardiographic PR and QRS intervals. No patient had right or left bundle branch block.

**Table 1:** Demographic and ECG data.

	SCN5A positive(n = 40)	SCN5A negative (n = 98)	Volunteers(n = 18)	P value* SCN5A positive vs.SCN5A negative
Age, years	45.1±14.3	43.9±12.5	42.0±8.7	0.77
Sex, man/woman (n)	22/18	49/49	8/10	0.46
Type of SCN5A mutation,missense/truncation (n)	33/7			
ECG parameters				
Heart rate, beats per min	64.7±10.1	68.8±11.0		0.049
PR, ms	192.2±30.5	162.3±23.4		<0.001
QRS, ms	110.7±15.2	100.7±11.7		<0.001
QT, ms	383.9±26.5	365.3±30.7		0.001
QTc, ms	396.3±28.3	387.9±24.2		0.09
S duration in II, ms	41.2±27.2	35.2±19.8		0.16
S amplitude in II, mV	0.23±0.23	0.19±0.15		0.23
S duration in III, ms	33.5±32.0	34.3±26.0		0.88
S amplitude in III, mV	0.18±0.24	0.21±0.26		0.46

* P value calculated with two-tailed t-test.

### Qualitative CMR Analysis

Only few patients had some evidence for myocardial fibrosis or fatty infiltration, and the proportion of such patients did not differ statistically significantly between the groups: fibrosis in 3 of 40 *SCN5A*-mut-positive and 3 of 98 *SCN5A*-mut-negative patients (p = 0.1); fatty infiltration in 1 of 40 *SCN5A*-mut-positive and 2 of 98 *SCN5A*-mut-negative patients (p = 0.9). No patient had evidence for myocardial edema.

### Quantitative CMR Analysis

#### Right ventricle

*SCN5A*-mut-positive patients had larger RV dimensions than subjects without *SCN5A* mutations (*SCN5A*-mut-negative patients or volunteers), as evidenced by larger RV end-systolic and end-diastolic volumes. Moreover, their RV ejection fractions were lower, albeit still in the normal range, than in volunteers (Table 2). In contrast, RVOT areas were significantly larger in both patient groups (*SCN5A*-mut-positive or *SCN5A*-mut-negative) than in volunteers. All other RV dimensions were similar between the 3 groups.

**Table 2:** CMR data of right and left ventricle.

CMR parameter	all patients (N = 138)	*SCN5A* positive (n = 40)	*SCN5A* negative (n = 98)	Volunteers (n = 18)	P value* all patients vs. volunteers	P value* *SCN5A* positive vs. *SCN5A* negative	P value* *SCN5A* positive vs. volunteers
Right ventricle							
RVEDV/BSA, ml/m^2^	83.9±15.2	88.0±14.4	82.4±14.9	74.8±24.2	0.030	0.041	0.019
RVESV/BSA, ml/m^2^	40.0±10.9	42.8±11.0	38.2±10.0	26.2±14.8	<0.001	0.015	<0.001
RVOT/BSA, cm^2^/m^2^	5.0±1.0	5.2±1.2	4.9±0.9	3.2±0.8	<0.001	0.140	<0.001
RVEF, %	52.7±7.8	52.0±6.0	54.1±6.9	66.7±9.8	<0.001	0.102	<0.001
Left ventricle							
LVEDV/BSA, ml/m^2^	80.0±13.9	83.4±16.3	78.7±12.7	72.3±19.3	0.059	0.107	0.049
LVESV/BSA, ml/m^2^	34.6±9.1	37.5±9.4	33.4±8.7	22.8±10.7	<0.001	0.025	<0.001
SWT, mm	8.8±1.7	9.6±1.7	9.6±1.8	9.2±0.5	0.754	0.902	0.651
PWT, mm	9.6±1.8	8.8±1.8	8.8±1.7	8.7±1.6	<0.903	0.965	<0.956
LVEF, %	56.9±7.4	54.9±6.5	57.8±7.6	69.2±9.1	<0.001	0.037	<0.001

LVEDV/BSA, left ventricular end-diastolic volume corrected for body surface area; LVEF, left ventricular ejection fraction; LVESV/BSA, left ventricular end-systolic volume corrected for body surface area; PWT, posterior wall thickness; RVEDV/BSA, right ventricular end-diastolic volume corrected for body surface area; RVEF, right ventricular ejection fraction; RVESV/BSA, right ventricular end-systolic volume corrected for body surface area; RVOT/BSA, right ventricular outflow tract area corrected for body surface area; SWT, septal wall thickness.

* P value calculated with linear regression analysis, corrected for sex and presence of coronary artery disease.

#### Left ventricle

*SCN5A*-mut-positive patients also had larger LV end-systolic volumes and lower (within the normal range) LV ejection fractions than subjects without *SCN5A* mutations (*SCN5A*-mut-negative patients or volunteers, Table 2). All other LV dimensions were similar between the 3 groups.

### Correlation between CMR Changes and Severity of Reduction in Na_v_1.5 Current

Having found that *SCN5A* mutations are associated with changes in ventricular dimensions and contractility, we studied whether the severity of CMR changes correlated with the severity of reduction in Na_v_1.5 current. As a measure of such reduction, we used the magnitude of PR or QRS prolongation. We found that PR and QRS width correlated statistically significantly with end-systolic volumes of RV and LV (Table 3). We also studied whether RV/LV dimensions and ejection fractions were lower in *SCN5A*-mut-positive patients with truncating *SCN5A* mutations (more reduction in Na_v_1.5 current predicted, n = 7) than in those with missense *SCN5A* mutations (less reduction in Na_v_1.5 current predicted, n = 33). Although RV and LV ejection fractions tended to be lower in the small group of patients with truncating mutations than in patients with missense mutations, these differences did not reach statistical significance (Table 4). Finally, we conducted a subanalysis in the 2 patients in this cohort (2 men, aged 25 and 38 years) who were compound heterozygous carriers of a *SCN5A* mutation. These patients appeared to have, on average, more RV and LV dilatation (RVEDV/BSA 107.5±0.9, RVESV/BSA 60.0±5.7, LVEDV/BSA 99.4±1.6, LVESV/BSA 50.0±3.7) and more reduced RVEF and LVEF (RVEF 44.1±5.8, LVEF 49.7±4.5) than patients with a single *SCN5A* mutation. Because these were only 2 patients, we conducted no statistical analysis to test for differences with patients with a single *SCN5A* mutation.

**Table 3:** Correlation between electrocardiographic PR and QRS width and end-systolic volumes of RV and LV.

	correlationcoefficient, R	P value*
PR vs. RVESV	0.21	0.014
QRS vs. RVESV	0.24	0.006
PR vs. LVESV	0.25	0.005
QRS vs. LVESV	0.26	0.003

LVESV, left ventricular end-systolic volume; RVESV, right ventricular end-systolic volume.

* P value calculated with linear regression analysis.

**Table 4:** RV/LV dimensions and ejection fractions in patients with truncating *SCN5A* mutations and missense *SCN5A* mutations.

	truncating mutation (n = 7)	missense mutation (n = 33)	P value*
Age, years	43.0±21.2	45.5±12.8	0.06
Sex, man/woman (n)	3/4	19/14	0.48
RVEDV/BSA, ml/m^2^	85.4±10.9	88.5±15.1	0.61
RVESV/BSA, ml/m^2^	44.4±9.6	42.5±11.4	0.68
RVEF, %	48.7±5.3	52.7±6.0	0.11
LVEDV/BSA, ml/m^2^	78.4±22.7	84.3±14.8	0.39
LVESV/BSA, ml/m^2^	35.4±8.7	38.0±9.6	0.51
LVEF, %	53.2±8.7	55.2±6.0	0.46

LVEDV/BSA, left ventricular end-diastolic volume corrected for body surface area; LVEF, left ventricular ejection fraction; LVESV/BSA, left ventricular end-systolic volume corrected for body surface area; RVEDV/BSA, right ventricular end-diastolic volume corrected for body surface area; RVEF, right ventricular ejection fraction; RVESV/BSA, right ventricular end-systolic volume corrected for body surface area.

* P value calculated with two-tailed t-test.

### Analysis of Possible Confounders

We studied the possible role of confounders for RV/LV contractile function. First, we studied for differences in the prevalence of a spontaneous type-1 pattern on the baseline ECG [21]. Among *SCN5A*-mut-positive patients, 10 had a spontaneous type-1 pattern on the baseline ECG, while 30 had a type-1 pattern only after ajmaline testing. These numbers were 7 and 91, respectively, for *SCN5A*-mut-negative patients (the number of ECGs per patient were 7.3±4.6 and 7.4±4.4 among *SCN5A*-mut-positive and *SCN5A*-mut-negative patients, respectively). The proportion of patients with a spontaneous type-1 pattern was statistically significantly higher among *SCN5A*-mut-positive patients (p = 0.004). It is conceivable that the higher proportion of such patients may indicate that *SCN5A*-mut-positive patients were more severely affected, and that this may (partly) explain the difference in RV and LV dimensions between *SCN5A*-mut-positive and *SCN5A*-mut-negative patients [15]. Yet, patients with a spontaneous type-1 pattern were not statistically significantly different from patients with a type-1 pattern only after ajmaline testing with regards to RVEDV, RVESV, LVEDV or LVESV.

The prevalences of hypertension or diabetes were not statistically significantly different between *SCN5A*-mut-positive patients (hypertension: 5/40, diabetes: 1/40) and *SCN5A*-mut-negative patients (hypertension: 14/98 [p = 0.8 vs. 5/40], diabetes: 5/98 [p = 0.5 vs. 1/40]). The prevalence of coronary artery disease was statistically significantly higher among *SCN5A*-mut-positive patients (4/40) than among *SCN5A*-mut-negative patients (1/98 [p = 0.01]). Yet, it is unlikely that the higher proportion of patients with coronary artery disease explained the higher values of RVEDV, RVESV, LVEDV or LVESV observed among *SCN5A*-mut-positive patients, because we corrected for the presence of coronary artery disease (and sex) when we compared these measures between the groups. Moreover, the average values for RVEDV, RVESV, LVEDV, and LVESV did not differ statistically significantly between patients with coronary artery disease and those without.

Heart rates of *SCN5A*-mut-positive patients were statistically significantly lower than in *SCN5A*-mut-negative patients during cardiac MRI examination (66.3±10.8 vs. 72.3±11.9 beats per minute, p = 0.007). Longer diastolic filling times may have contributed in part to larger end-diastolic volumes of RV and LV in *SCN5A*-mut-positive patients. Indeed, we found that cycle length correlated statistically significantly with RVEDV/BSA (p = 0.002), RVESV/BSA (p = 0.003), LVEDV/BSA (p = 0.001), and LVESV/BSA (p = 0.02) (but not with RVEF [p = 0.2] or LVEF [p = 0.9]). Yet, these correlations were only weak (correlation coefficients were 0.27, 0.25, 0.28, and 0.20, respectively), suggesting that this is not the only factor to explain differences in RV and LV dimensions between *SCN5A*-mut-positive and *SCN5A*-mut-negative patients.

## Discussion

We found that BrS patients with an *SCN5A* mutation have enlargement of both RV and LV, compared with persons without an *SCN5A* mutation (*SCN5A*-mut-negative BrS patients or volunteers). The severity of RV/LV enlargement correlated with the magnitude of reduction in Na_v_1.5 current, suggesting a causative role of reduction in Na_v_1.5 current. However, this correlation was not strong. Previous studies add further evidence to the notion that Na_v_1.5 current reduction alone is not sufficient to cause structural changes in RV and LV. For instance, rats and mice with chronically reduced Na_v_1.5 current secondary to long-term (18–24 months) treatment with flecainide, a cardiac antiarrhythmic drug that blocks Na_v_1.5 current, did not develop cardiac fibrosis [22]. Thus, the presence of abnormal Na_v_1.5 proteins *per se* in our *SCN5A*-mut-positive patients may have contributed to the increases in cardiac dimensions and reductions in contractility. This finding would support the recent proposal that Na_v_1.5 is involved in maintaining structural integrity of the heart. This proposal is supported by various transgenic mouse studies in which *SCN5A* was mutated to recapitulate loss-of-function *SCN5A* channelopathy exhibited age-dependent degenerative histopathologic changes, including fibrosis and fatty replacement [23]–[24].

Na_v_1.5 is a transmembrane protein composed of the main pore-forming α-subunit and two subsidiary β-subunits (β_1_ and β_2_) [1]. There is accumulating evidence that Na_v_1.5 forms part of a macromolecular complex [7] and that its function is modulated by cytoskeleton proteins, e.g., tubulin [25], syntrophin, and dystrophin [26]–[27]. Given these interactions between Na_v_1.5 and cytoskeleton proteins, it is conceivable that, conversely, abnormal Na_v_1.5 proteins affect cytoskeleton function and structural integrity of cardiomyocytes. Yet, this proposal awaits experimental evidence.

While we found that ejection fractions of RV and LV were lower in carriers of a *SCN5A* mutation than in non-carriers, RVOT diameters were similarly increased in *SCN5A*-mut-positive and *SCN5A*-mut-negative patients. This finding indicates that structural derangement of RV is a common feature of BrS. RV may be more susceptible than LV to stressors that disrupt structural integrity. Support for this notion comes from the observation that fibrosis was largest in RV, in particular, RVOT, in control hearts. The biological basis of these observations is a matter of speculation. It may lie in fundamental differences in gene expression profiles between RV and LV that can be traced to embryologic development of the heart [28]. Thus, electrical and mechanical properties of RV and LV are intrinsically different, and genetic and environmental stressors may act differently in RV and LV. Importantly, *SCN5A* expression is higher in RV than in LV [29]. Thus, if Na_v_1.5 contributes to cytokeleton integrity of the heart, loss-of-function *SCN5A* channelopathy is expected to affect RV more strongly than LV. Still, we obtained evidence that the pathophysiologic derangements in BrS are not confined to RV, as long assumed, but that LV is also significantly affected, as recently reported [15]. The observation that BrS affects both ventricles, and that RVOT dilatation is a feature shared by all BrS patients, regardless of the presence of a *SCN5A* mutation, may be taken as supportive evidence for the notion that BrS should be considered a cardiomyopathy with predominant but not exclusive involvement of RV, similar to arrhythmogenic right ventricular cardiomyopathy. Moreover, it suggests that the disease-causing genes in BrS patients who have no *SCN5A* mutation are also involved in structural integrity of the heart, similar to *SCN5A*. One explanation could be that the protein products of these genes (most of which await discovery) also interact with the cytoskeleton, possibly through their interaction with *SCN5A*. Indeed, some known genes that are involved in BrS (although rarely) interact with *SCN5A*, notably *SCN1B* [30] and *GPD1-L* [31]. It is conceivable that the *SCN5A*-mut-negative BrS patients in our study carried mutations in such genes (but not in *SCN1B* and *GPD1-L*, which were screened), and that the presence of these mutations explained, at least in part, why *SCN5A*-mut-positive patients differed far less from *SCN5A*-mut-negative patients than from controls with regards to CMR parameters. Similarly, the differences between *SCN5A*-mut-positive and *SCN5A*-mut-negative patients were only statistically significant for RVEDV, RVESV, LVESV, and LVEF, and we observed no differences in the incidence of fibrosis or fatty infiltration between both groups. While the lack of statistical significance for fibrosis or fatty infiltration may indicate a lack of statistical power in our study, it is to be expected that differences between *SCN5A*-mut-positive and *SCN5A*-mut-negative patients are relatively small, and that other parameters for structural properties are not different between both groups, given the fact that Brugada syndrome is generally regarded a primary electrical disease, i.e., a disease in which gross structural changes cannot be routinely detected with current cardiac imaging methods.

Our findings of larger RV dimensions, reduced contractile function of both RV and LV, and larger RVOT areas in BrS patients are in accordance with previous studies [14]–[17]. However, some of our values (e.g., RVEDV and LVEDV) are different from those studies. Since our values of end-diastolic volumes, end-systolic volumes, and ejection fractions of LV and RV are within the range of published normalized data [32], we believe that these differences can be mainly attributed to methodological differences (e.g., determining which basal slice to include in the analysis). Of importance, we included our own cohort of healthy volunteers in which the analysis was done in the same way as in the patients.

While we found that *SCN5A*-mut-positive BrS have reduced ejection fractions, previous studies have indicated that BrS patients also have slow conduction of the cardiac electrical impulse, notably in RV [33]–[34]. We cannot rule out that conduction slowing may have resulted in loss of contractile synchrony between various regions of the heart [35], and that this may have contributed partly to reduced ejection fractions. In any case, patients with right or left bundle branch block were not included in the present analysis.

Moreover, we found that QRS (and PR) width correlated with end-systolic volumes of RV and LV. Yet, it must be noted that QRS width *per se* may not reflect solely sodium channel malfunction/deficiency, but that it may also be caused by other factors, e.g., nonsynchronous activation. Clearly, ECG analysis alone cannot distinguish between the effects of each of these (and possibly other) factors. Still, nonsynchronous activation may be an important factor that may adversely affect RV and LV hemodynamics. This has been clearly shown in numerous studies that demonstrated the beneficial effects of cardiac resynchronization therapy for patients with left ventricular failure which is associated with dyssynchronous activation of parts of the LV [36]. However, in the case of BrS, a disease that predominantly affects the RV, dyssynchrony is more likely to relate to later activation of RV with respect to LV, rather than between various parts of LV [35]. Moreover, we recently showed not only that, in RV disease, RV activation is delayed with respect to LV activation, and that this delay is associated with adverse hemodynamic effects, but also that these adverse effects can be corrected by resynchronization (pacing) of RV [37].

### Limitations

Cardiac dilatation and reduced contractility may be due to histologic changes (fibrosis, fatty degeneration), as recently reported [12]. However, we found virtually no signs for these abnormalities. It is probable that the spatial resolution of current clinical imaging methods, such as CMR, is too low to detect such changes; this would explain why BrS has been classified a primary electrical disease, and it has taken long before it was recognized that structural derangements are also present. We expect that the ability to study larger cohorts or use new imaging techniques may unmask differences in the incidence of histopathologic abnormalities between *SCN5A*-mut-positive and *SCN5A*-mut-negative patients and/or between BrS patients and non-BrS patients. It is conceivable that such an ability may have immediate clinical implications, e.g., for risk stratification. For instance, studies are now emerging which clearly demonstrate that areas of fibrosis are crucially linked to the occurrence/inducibility of reentrant arrhythmias, including ventricular fibrillation, in BrS patients [9]–[10], [38].

While this study focused on a possible role of Na_v_1.5 in determining RV and LV dimensions and contractility, it is conceivable that other sarcolemmal ion channels involved in BrS, e.g., L-type calcium channels may also play a role; the L-type calcium channel encoding genes implicated in BrS [39] were, however, not screened.

### Conclusions

Loss-of-function *SCN5A* mutations are associated with dilatation and impairment in contractile function of both ventricles that can be detected by CMR analysis. These findings support the notion that Na_v_1.5 is involved in maintaining structural integrity of the heart.
